# Supplementary material for: Formation and energetics of head-to-head and tail-to-tail domain walls in hafnium zirconium oxide
Source: Sci Rep. 2024 Apr 29;14:9861. doi: 10.1038/s41598-024-60155-x (PMC11058872; doi:10.1038/s41598-024-60155-x)
Supplement: Supplementary file 1 — Supplementary Figures. [file 41598_2024_60155_MOESM1_ESM.docx]

**Supplementary Information**

**Formation and Energetics of Head-to-Head and Tail-to-Tail Domain Walls in Hafnium Zirconium Oxide**

Tanmoy Kumar Paul, Atanu Kumar Saha, Sumeet Kumar Gupta

*Purdue University, West Lafayette, Indiana, 47907, USA*

*Email:* [*paul115@purdue.edu*](mailto:paul115@purdue.edu%20)  */ Phone: (765) 607-3147*


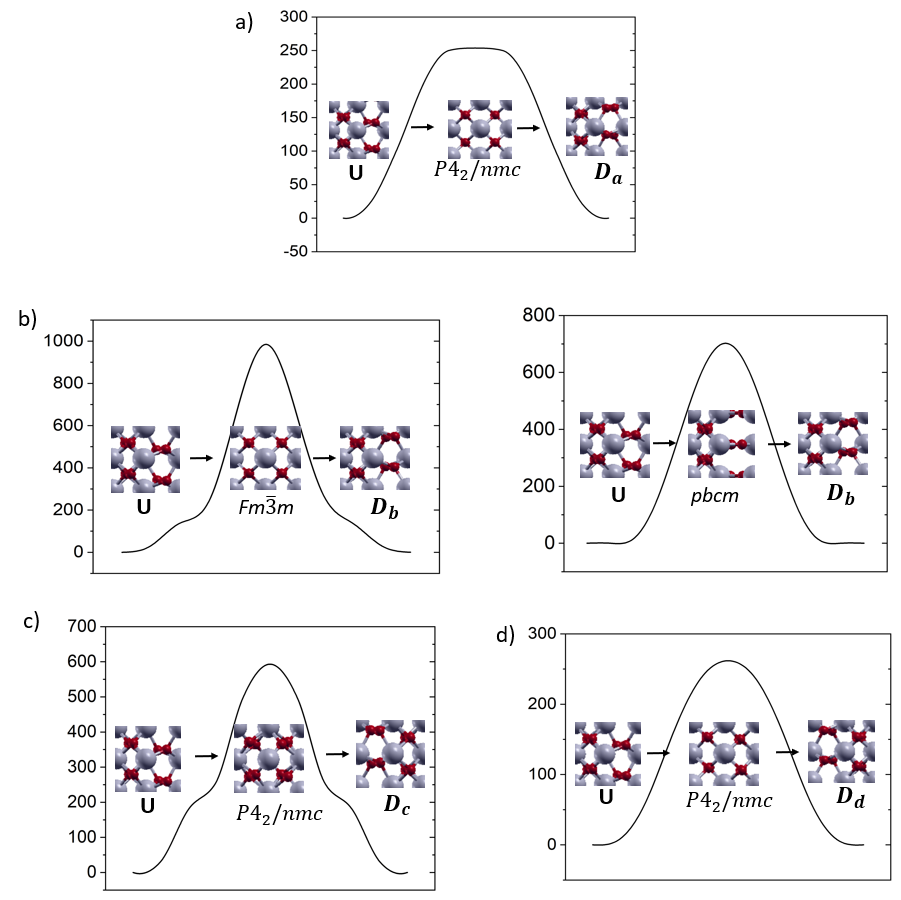


**Figure S1** a)-d) Polarization reversal path for upward polarized unit cell, U to downward polarized unit cells $D_{a}$, $D_{b}$, $D_{c}$ and $D_{d}$ respectively. The unit cells are taken from Figure 1 of main text. From U to $D_{b}$ transition, both *Fm*$\bar{3}$*m* and *pbcm* can occur as intermediate state as shown in the two paths of b). For U to $D_{a}$, $D_{c}$ and $D_{d}$ transition, $P4_{2}/nmc$ occurs as the intermediate phase. As evident from the figures, the view of the *xz* plane of intermediate $P4_{2}/nmc$ phase in S1c is different than the *xz* planes in S1a and S1d. It turns out that during NEB path reversal, the strain induced on the intermediate $P4_{2}/nmc$ phase in S1c is much higher compared to S1a and S1d. Therefore, the energy of the intermediate $P4_{2}/nmc$ phase in Figure S1c is higher than the $P4_{2}/nmc$ phases in Figure S1a and S1d. Note that, if variable cell NEB calculation is performed, the energies of ${P4}_{2}/nmc$ phase in all cases should be similar. However, fixing the lattice parameter during switching calculation is a fairly viable assumption, if one considers fast switching.


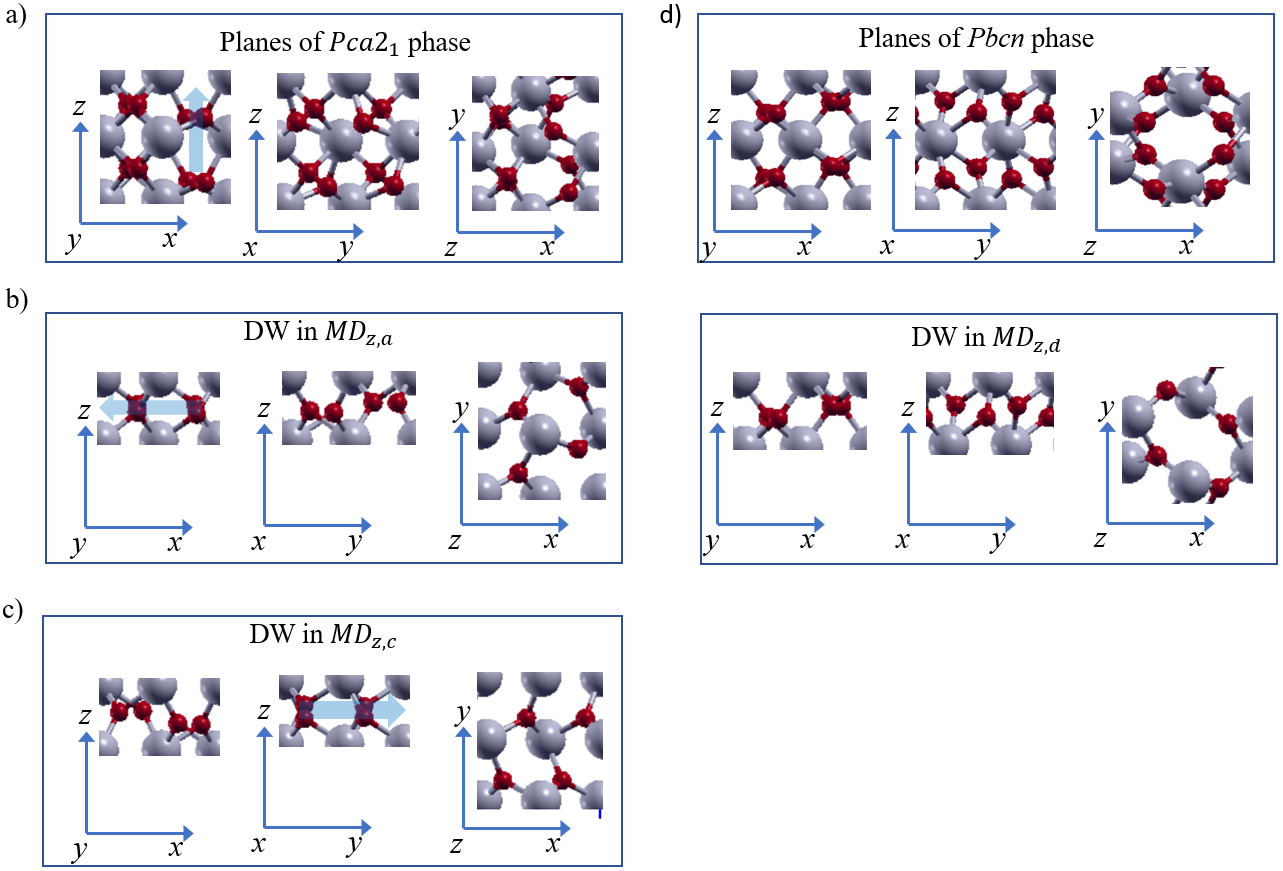


**Figure S2** Atomic configuration with views from three orthogonal planes. a) inside upward polarized domain of *Pca2_1_* phase b-d) within DWs in ${MD}_{z,a}$, ${MD}_{z,c}$ and ${MD}_{z,d}$ of Figure 2 respectively. All the DWs are half unit cell thick and so half-cell thick configuration is shown here. The DWs in ${MD}_{z,a}$ and ${MD}_{z,c}$ resemble orthorhombic *Pca2_1_* phase and are 90$^{\circ}$ rotated with respect to the domains. In the DW of ${MD}_{z,a}$, the polarization axis lies along the *x* direction. On the other hand, in the DW of ${MD}_{z,c}$, the polarization axis lies along the *y* direction. The DW in ${MD}_{z,d}$ resemble orthorhombic *pbcn* phase. For better comparison purpose, similar planes of full unit cell of *pbcn* phase are also demonstrated in S2d.

Inside both the upward and downward polarized domains, the lattice parameters along *x*, *y* and *z* direction are a (5.06 A°), b (5.28 A°), and c (5.09 A°), respectively. In ${MD}_{z,a}$, the DW lattice parameter along *x*, *y* and *z* direction are c (5.09 A°), b (5.28 A°) and a (5.06 A°), respectively, as can be inferred by comparing atomic structures in Figure S2a and S2b. Thus, although there is 90° rotation of the DW, the strain induced at the DW is very less (because of the small difference in the magnitude of lattice parameter a and c). On the other hand, in ${MD}_{z,c}$, the DW lattice parameter along *x*, *y* and *z* direction are b (5.28 A°), c (5.09 A°), and a (5.06 A°), respectively, as can be inferred by comparing atomic structures in Figure S2a and S2c. Thus, although the domains are at cross-pattern in ${MD}_{z,c}$, larger strain is induced in the DW compared to ${MD}_{z,a}$(because of the larger magnitude of lattice parameter b). This causes ${MD}_{z,c}$ to be energetically the costliest.

| 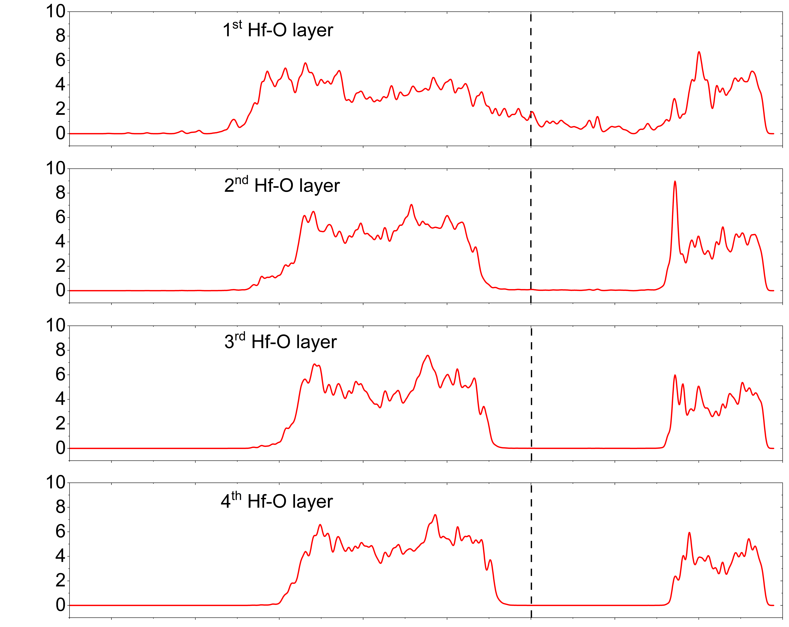  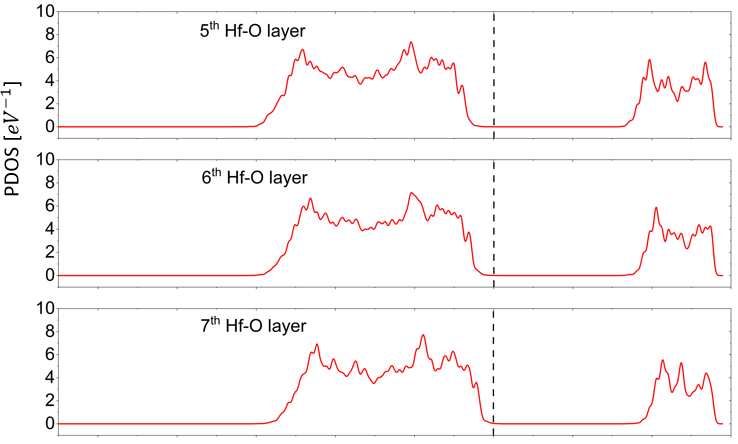  VB shifts towards fermi level  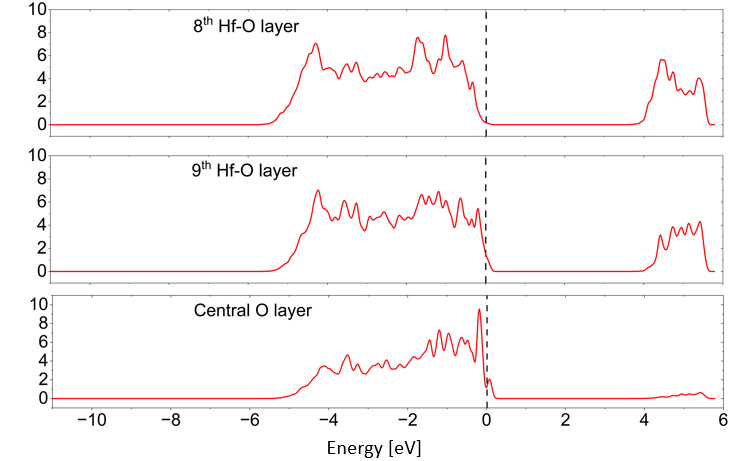 | 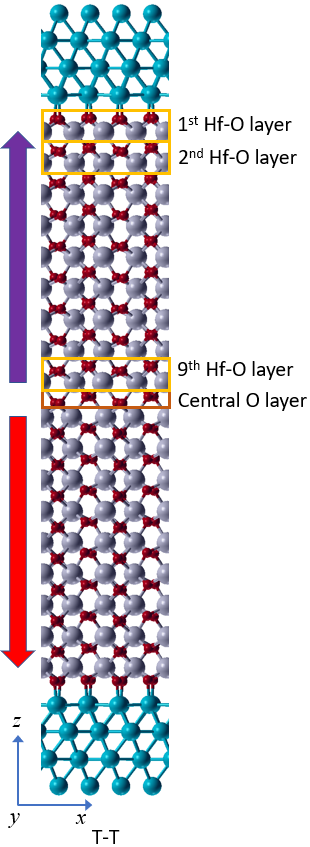 |
| --- | --- |

**Figure S3** Layer wise PDOS from the interfacial Hf-O atoms towards the central O atoms at the DW for T-T configuration. VB shifts towards the fermi level as we move towards the DW region which indicates that holes appear near the DW. (The first Hf-O layer is interfacial; therefore, they are metalized and corresponding projected bandgap is not observable.)


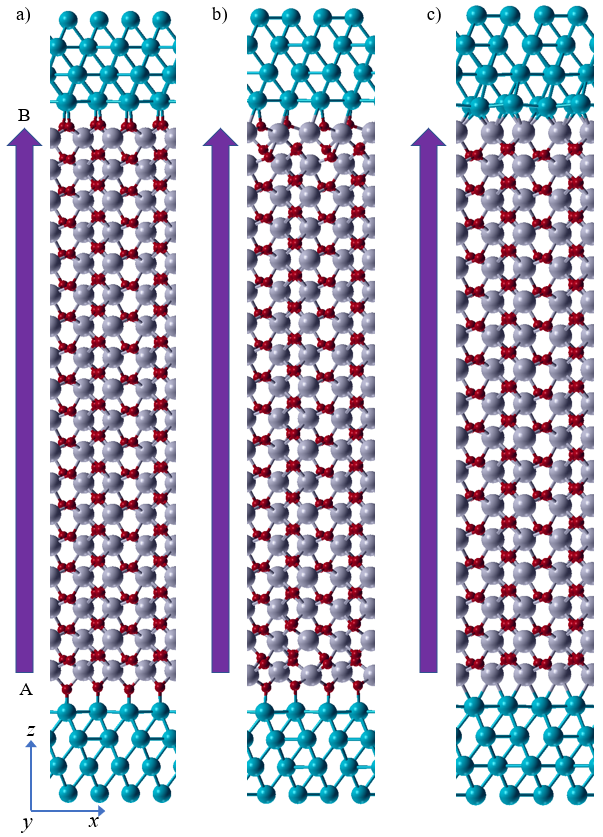


**Figure S4** Atomic configuration of single domain structures with a) vacancy-free O-ended interfaces b) interfaces with one spacer and one polar vacancy c) Hf/Zr-ended interfaces. A and B interfaces are marked as bottom and top interfaces respectively.


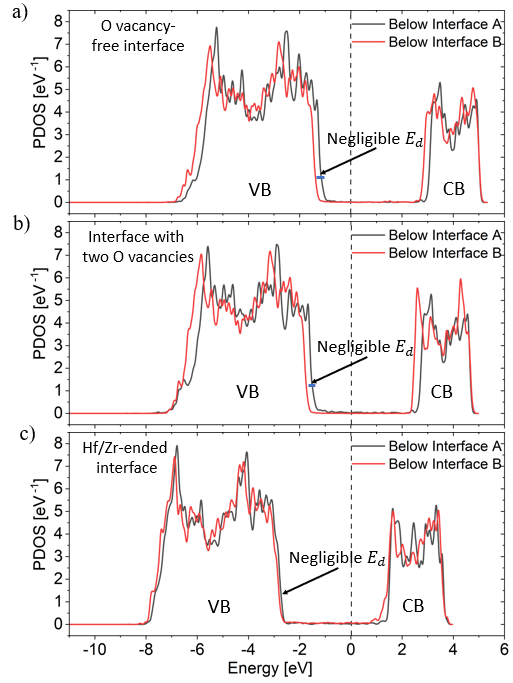


**Figure S5** Projected Density of States (PDOS) of atomic layers of SD HZO adjacent to the two interfaces for a) O-ended interfaces without vacancy b) interfaces with one spacer and one polar vacancy c) Hf/Zr-ended interfaces shown in Figure S4a-c respectively. The negligible shift between the bands near the two interfaces dictates negligible depolarization field, i.e., excellent bound charge screening by metal, irrespective of oxygen vacancy density.


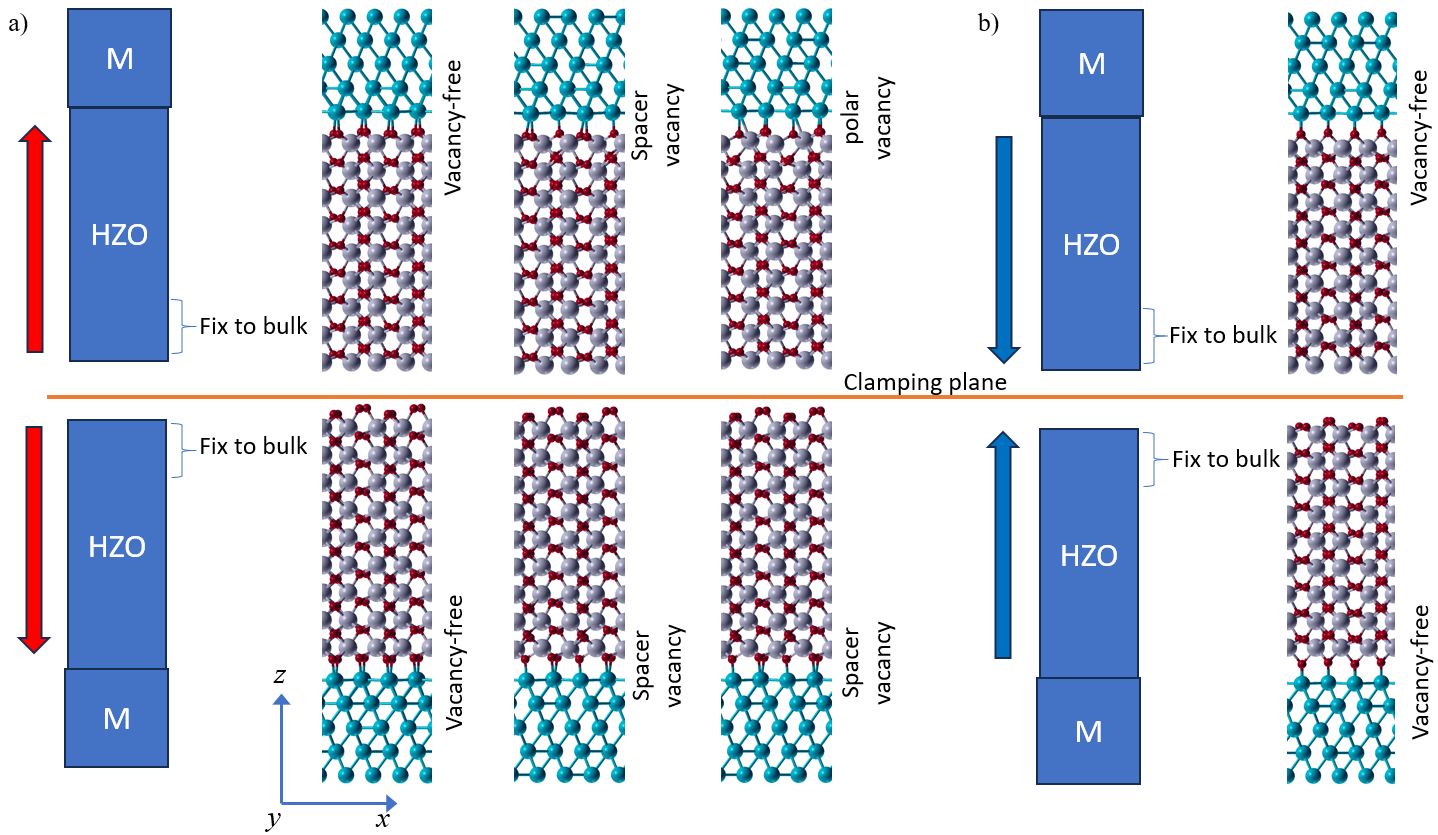


**Figure S6** a) Metal-HZO stacks to form T-T configuration for vacancy-free O-ended interface and interfaces with single oxygen vacancy in either polar or spacer layer. b) Metal-HZO stacks to form H-H configuration for vacancy-free O-ended interface. To obtain relaxed interfaces of each stack, the furthest HZO unit cell from each of the interfaces are kept fixed to bulk atomic configurations. The in-plane lattice parameters are kept fixed to HZO and out-of-plane lattice parameter is relaxed with the presence of a vacuum on both sides. The top and bottom stacks are clamped together in the orange plane to get initial (not fully relaxed) structures of T-T and H-H configurations.


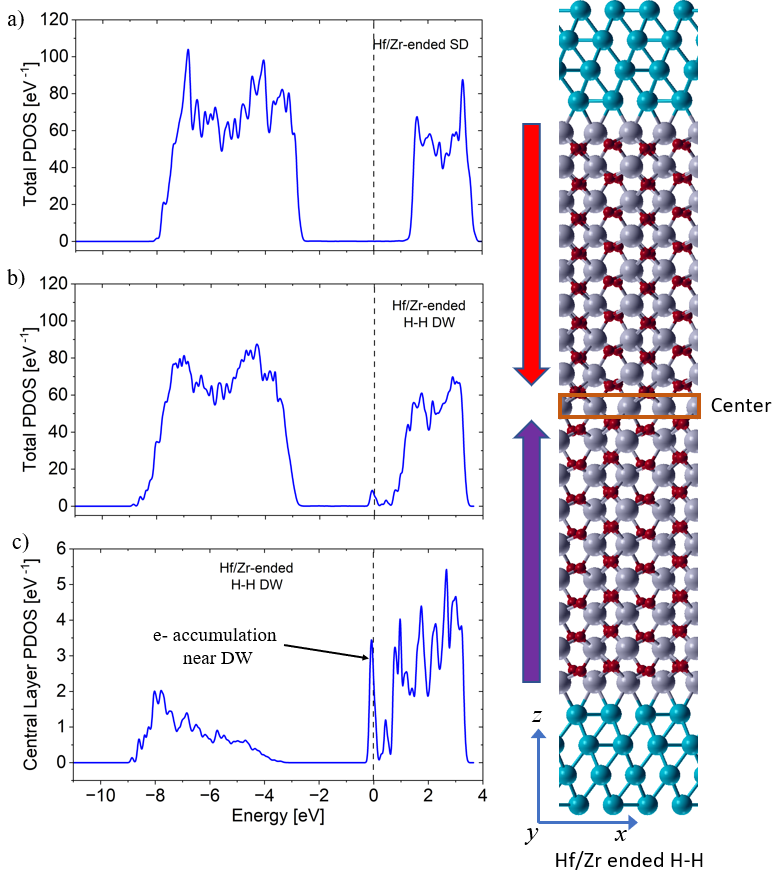


**Figure S7** Total PDOS of atomic layers of a) SD HZO and b) MD HZO of H-H configuration for Hf/Zr-ended interfaces c) PDOS at central Hf/Zr layer for H-H DW configuration in HZO. The CB minimum and fermi level suggest that electrons appear at the DW to screen positive polarization bound charges. This comes at the cost of depolarization field inside the domains. The structure at the right side shows the abovementioned H-H structure.
